# Computational fluid dynamics simulation of the upper airway response to large incisor retraction in adult class I bimaxillary protrusion patients

**DOI:** 10.1038/srep45706

**Published:** 2017-04-07

**Authors:** Zhe Zheng, Hong Liu, Qi Xu, Wei Wu, Liling Du, Hong Chen, Yiwen Zhang, Dongxu Liu

**Affiliations:** 1 Department of Orthodontics, College of Stomatology, Shandong University, Shandong Provincial Key Laboratory of Oral Tissue Regeneration, Jinan, 250012, China; 2 Department of Stomatology, Weifang People’s Hospital, Weifang, 261041, China

## Abstract

The changes of the upper airway after large retraction of the incisors in adult class I bimaxillary protrusion patients were assessed mainly focused on the anatomic variation and ignored the functional changes. This study aimed to investigate the changes of the upper airway in adult class I bimaxillary protrusion patients after extraction treatment using the functional images based on computational fluid dynamics (CFD). CFD was implemented after 3D reconstruction based on the CBCT of 30 patients who have completed extraction treatment. After treatment, pressure drop in the minimum area, oropharynx, and hypopharynx increased significantly. The minimum pressure and the maximum velocity mainly located in the hypopharynx in pre-treatment while they mostly occured in the oropharynx after treatment. Statistically significant correlation between pressure drop and anatomic parameters, pressure drop and treatment outcomes was found. No statistical significance changes in pressure drop and volume of nasopharynx was found. This study suggested that the risk of pharyngeal collapsing become higher after extraction treatment with maximum anchorage in bimaxillary protrusion adult patients. Those adverse changes should be taken into consideration especially for high-risk patients to avoid undesired weakening of the respiratory function in clinical treatment.

A constrictive airway may influence respiratory function, and induce symptoms of obstructive sleep apnea syndrome (OSAS), which affects quality of life and may be life-threatening^1^,^2^. Many studies have demonstrated that orthodontic treatment causes morphological or functional changes of upper airway^3^,^4^. Therefore, detailed evaluation of the upper airway is essential for orthodontic diagnosis and planning.

Bimaxillary protrusion is a common malocclusion in China. Due to the inharmonic facial profile, Patients with bimaxillary protrusion are seeking for orthodontic treatment to improve their overly prominent upper and lower incisors and lips. A popular orthodontic treatment for bimaxillary protrusion includes extraction of four first premolars combining with the retraction of anterior teeth using maximum anchorage, which help the patients achieve resultant decrease in soft tissue procumbency and convexity. The relationship between incisors retraction and lip procumbency has been well recognized^5^. However, the changes of the upper airway in adult class I bimaxillary protrusion patients after retraction of the large incisors remains to be controversial.

Previous studies have investigated the effects of extraction treatment on upper airway in bimaxillary protrusion patients using two-dimensional (2D) cephalometric measurements or three-dimensional (3D) CBCT measurements^6^,^7^,^8^,^9^. Some studies found that the middle and inferior airway dimensions diminished after the extraction treatment^6^,^7^,^9^, while Al Maaitah *et al*.^8^ found no difference in the upper airway between pre and post-treatment. All of these studies were designed to explore the morphological changes in 2D or 3D images, and ignored the potential functional changes. Wootton DM *et al*.’s work suggested that CFD model endpoints based on pressure drops in the pharynx were more closely associated with the presence and severity of OSAS than anatomical endpoints, which may be a better tool to predict the risk of the upper airway collapsing^10^.

Computational Fluid Dynamics (CFD) is a validated method to accurately compute aerodynamic flow characteristics of the upper airway^11^,^12^,^13^,^14^,^15^, which allows describing regional flow and pressure profiles in the upper airway. Literature had reported that a CFD model is more sensitive or accurate than anatomical parameters alone in evaluating the effects of airway restriction in OSAS^10^. Therefore, CFD simulation together with 3D upper airway model makes it possible to provide a more comprehensive understanding of the variety in pharynx after extraction treatment.

The aim of this study is to evaluate the pharyngeal airflow response to large incisor retraction in class I adult bimaxillary protrusion patients using CFD simulation based on the patient-specific CBCT dataset.

## Materials and Methods

### Subjects

The study was reviewed and approved by School of Stomatology Shandong University Research Ethic Board (protocol number 20160103). All the written informed consents were received from the patients, and the study was conducted according to the tenets of the Declaration of Helsinki for research involving human subjects. The methods were carried out in accordance with the approved guidelines of SCIENTIFIC REPORTS. The sample of this study consisted 30 (19 females, 11 males, age 25.87 ± 0.78 years, BMI 20.56 ± 1.48 kg/m^2^) class I bimaxillary dentoalveolar protrusion patients, who were treated by extraction of four first premolars using maximum anchorage. The exclusion criteria included: (1) patients have any other severe craniofacial anomalies except class I bimaxillary dentoalveolar protrusion, (2) age under 18 years old, (3) a history of serious diseases, or acquiring serious diseases during treament, such as serious cardiovascular disease, respiratory system disease, serious craniofacial deformity, severe nasal diseases and relative surgical history, (4) weight increased apparently into obese (BMI > 30 kg/m^2^) after treatment. The patients were informed consent, including the potential risk associated with CT radiation and miniscrew methodologies. Oriental pre-adjusted appliance KOSAKA slot brackets (OPA-K, Tomy; Fukushima-ken, Japan) were used, and miniscrews were implanted as anchorage for the retraction and intrusion of the teeth. A force of 150 g per side of elastic chains was applied from the miniscrew to the upper crimpable hook to retract and intrude the upper anterior tooth. The patients were examined at 1-month intervals until the retraction of the upper and lower anterior teeth completed.

### Reconstruction of the model and mesh generation

**Figure 1:**
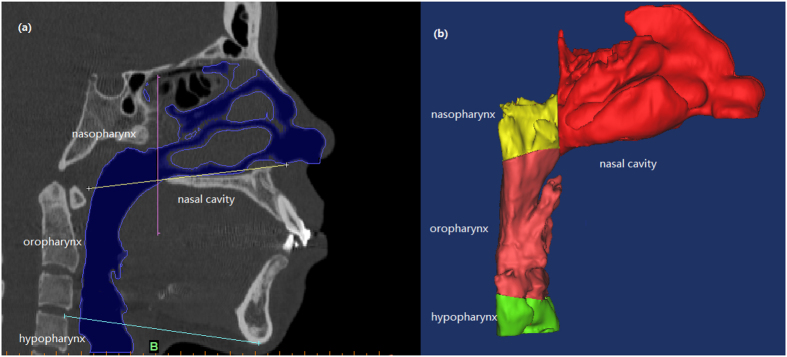
(**a**) The upper airway was divided into nasal cavity, nasoparynx, oropharynx, and hypopharynx and (**b**) 3D model of each section was reconstructed respectively.

The three-dimensional models used in the calculation were developed from pre- and post-treatment CBCT data from patients selected. All CBCT scans were implemented with each patient awake in the position of the Frankfort horizontal plane parallel to the floor using the same CBCT scanner (KaVo Dental GmbH, Bismarckring, Germany. scan time: 8.9 seconds; slice thickness: 0.4 mm, 120 kV, 5 mA) by the same operator. Patients were guided to close their mouths with the maximum intercuspation and the tongues touching their hard palates during the scanning. The pre- and post-treatment CBCT scans of patients were collected as T1 and T2 data. Subsequently, the dataset was exported in DICOM (Digital Imaging and Communications in Medicine) file format, and then was read into MIMICS16.0 (Materialism’s Interactive Medical Image Control System) software. After that, the models were reorientated in three planes: in the coronal view, the most inferior points on the infraorbital margin (orbitale) lie on the same horizontal plane; in the sagittal view, the models were reorientated to make the Frankfort plane horizontal; in the axial view, the models were reorientated to make the line through the crista galli and the midpoint on the anterior margin of foramen magnum (basion) vertical^16^. The upper airway of interest was segmented by setting the threshold between −1024 Hounsfield Units (HU) and −480 HU, and the 3D anatomically accurate patient-specific models were reconstructed. An appropriate smoothing algorithm was used to transform the 3D model into a smooth one without the loss of the patient-specific characters in the shape of the upper airway (Fig. 1). After that, the stereolithography (STL) files of the 3D models were imported into ANSYS ICEM CFD (ANSYS 16.0) for model repairing and mesh generating.

**Figure 2:**
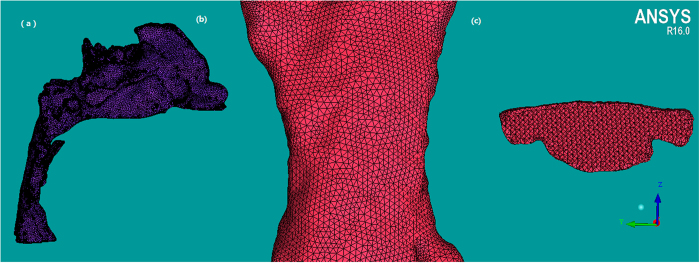
Mesh generation of the upper airway 3D geometry in (**a**) overall, (**b**) amplified, and (**c**) cross-sectional view.

**Figure 3:**
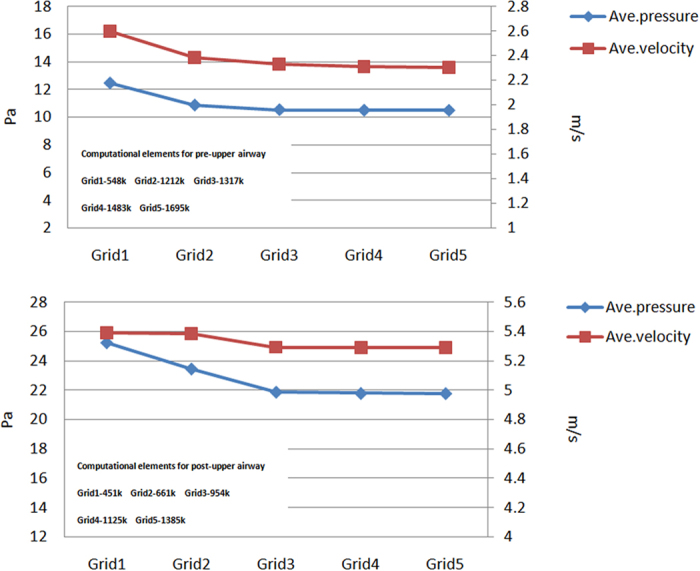
Effects of computational cell size on calculated results, changes of average pressure and average velocity on the selected plane in a (**a**) pre-treatment model and (**b**) post-treatment model.

Unstructured tetrahedral volume mesh was generated in ANSYS ICEM CFD (ANSYS 16.0) using hybrid mesh scheme (Fig. 2). A grid convergence analysis was performed by repeating the solution with five different element size meshes (Grid 1, Grid 2, Grid 3, Grid 4 and Grid5) to establish grid independence solutions. Similar element size was used in the pre-reatment and post-treatment models for reliable comparisons of the results. Changes in the average pressure and average velocity in a selected plane were used as convergence criteria. when the changes in those criteria were less than 1%, an acceptable level of grid-independence was achieved. As shown in Fig. 3, changes of the average pressure and average velocity between Grid 3 and Grid 4 were 0.32%, 0.93% for pretreatment model, and 0.38%, 0.02% for post-treatment model. It was suggested that changes in those criteria become practically negligible if Grid3 is used. Thus, all the data presented in the current work were from a simulation with Grid3, resulting in a computational grid of pre- and post-treatment typically consisting of 1.10 ± 0.05 million and 1.01 ± 0.09 million elements, respectively. The maximum skewness of the grids was 0.87. Then the 3D models were imported into the Reynolds Average Navier Stokes CFD solver (Fluent 16.0, Fluent Inc.).

### Computational fluid dynamics

**Figure 4:**
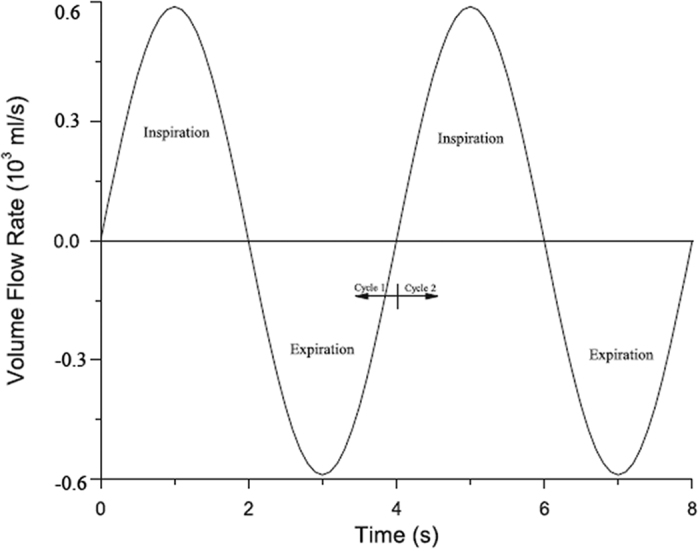
A typical flow input waveform for transient flow in a respiratory circle.

Flow simulations were performed using the Reynolds Average Navier Stokes CFD solver (Fluent 16.0, Fluent Inc.). Inspiratory upper airway flow was modeled, as inspiration is associated with negative pressures causing airway collapse in OSAS, greater airflow pressure and velocity gradients. Steady laminar flow field was applied in this study. Previous studies showed that fully developed turbulence would not be reached until a flow rate of 30 L/min^17^. The flow rate in the current study was less than 24 L/min, therefore the airflow was presumed as incompressible and laminar. Wootton *et al*.^11^ have validated steady model results with unsteady flow simulation, and the pressure distribution in the steady model at peak flow was very similar to the unsteady model, the most changes in pressure was less than 2 Pa. To reduce the computational resources needed, steady state condition was used in this study. The magnitude of the peak flow rate was calculated by the formula V = Q/A. Q was the volume flow rate of the model, and A was the area of the bottom of the hypopharynx. In this study, the respiratory tidal volume was 600 mL, and the respiratory cycle was T = 4 s, an approximate sinusoidal variation in the airflow rate with time was observed in the rhinomanometer test, and the ratio of the inspiratory time and expiratory time was 1:1 (Fig. 4), 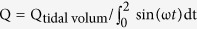, ω = π/2 is the angular frequency, Q_tidal volum_ = 600 ml. For inspiration process, an ambient static pressure condition was set at the nostril (the flow outlet), the negative value of velocity at the peak flow rate was set at the bottom of the hypopharynx (the flow inlet), the wall surface of the geometry was assumed to be non-slip.

The effects of some factors such as the temperature, humidity and vibrissae on the fluid were neglected in the simulation, while the gravity was included. The calculation residual was set as 10^−4^ and the iteration numbers were 1000 steps when all residuals fell below 0.2%. Second-order discretization schemes were used with pressure-velocity coupling solved through the SIMPLE scheme.

### Outcome parameters

Two planes: a line passing from anterior nasal spine to posterior nasal spine and a line passing from the anterosuperior edge of the fourth cervical vertebra (C4) to menton, which have been proved to be reliable and reproducible landmarks for defining upper airway margins^16^ were uesd to divided the whole pharynx into three parts (nasopharynx, oropharynx, and hypopharynx). In this study, volumes of the whole pharynx (V) and each part of the pharynx: nasopharynx (V_na_), oropharynx (V_or_), and hypopharynx (V_hy_) was calculated. The minimum cross-sectional area (A_min_) and its location was defined. Parameters above were used to assess the size changes of the pharynx. In addition, the mean cross-sectional area (A_mean_) was computed by ratio of the pharyngeal volume and the length of the pharynx (V/L). A_min_/A_mean_ was also obtained to explore the uniform of the cross-sectional changes. Upper central incisor retraction amount at edge in the horizontal direction (ICE) were achieved through registration of the pre- and post-treatment models, and the process for registration was presented in details in the work of Chen Y *et al*.^7^.

Once the simulation was completed, all of the cross sections from choana to the bottom of hypopharynx were selected per millimeter along Z-axis for parameters calculation. Area-weighted average pressure and velocity in each cross-section was extracted to evaluate the location of the minimum area-weighted average pressure and the maximum velocity in the pharynx. The area-averaged pressure drop from choana to the minimum area (ΔP_max_) was defined to quantify the effect of the pharyngeal airway narrowing on the flow field. Airflow pressure drops of nasopharynx (ΔP_na_), oropharynx (ΔP_or_), and laryngopharynx (ΔP_hy_) were counted by ΔP = P_max_ − P_min_. The resistance of the airway (R) was achieved by the formula: R = Q/ΔP (Q was pharyngeal inlet volume flow rate). Because the flow rate was constant in our study, the ΔP reflected the changes of the resistance for each pharyngeal part.

All the parameters were measured twice by the same researcher, and the average value was applied to the study.

### Statistical analysis

All statistical analyses were performed using the SPSS 17.0 software package. For each variable themean and the standard deviation (SD) were calculated. The differences between the flow dynamics and anatomical parameters of the upper airway between T1 and T2 were analyzed by paired-t test, and the relationships between the ΔP and ICE were assessed using Pearson correlation coefficient. To assess the measurement errors, all the variables were measured twice with the manual method by the same operator with a 2-week interval. The error of method was calculated as follows: 
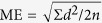
 (where d indicates deviations between the two measurements, and n indicates number of paired objects). Intraexaminer reliability was calculated by intraclass correlation coefficient (ICC) for the measurements obtained by the examiner at both times. For all the analyses, P < 0.05 was considered significant. Stepdown Bonferroni multiple testing was used to correct the P-values.

## Results

No significant difference between the first and repeated measurements were observed, which proved the reliability between the first and second measurements (ICC from0.92 to 0.97). The maximum ME of pharyngeal volume and area measurements were 0.15 mm^3^ and 0.31 mm^2^, respectively, while the maximum ME of pressure measurements was 0.47 Pa in the numerical simulation of pharyngeal airflow, and 0.24 mm of the upper central incisor retraction amount at edge in the horizontal direction.

A statistically significant decrease between the pre and post-treatment was observed in the volume and the value of the A_min_ and A_min_/A_mean_ (Table 1) of the oropharynx and hypopharynx, while the difference in the nasopharynx between pre and post-treatment was not significant. The location of the minimum of the cross-section moved upward from the hypopharynx to the oropharynx in the post-treatment patients compared to the pre-treatment patients. Upper central incisor retraction amount at edge in the horizontal direction (ICE) was 6.84 ± 1.68 mm after the extraction treatment.

**Figure 5:**
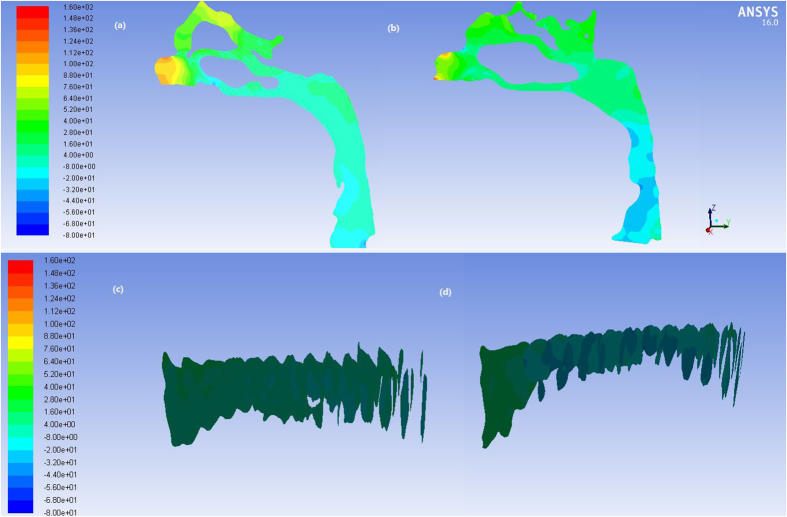
The pressure profile of the pharyngeal airflow in (**a**) pre- and (**b**) post-treatment, and the cross section every 5 mm along Z-axis in (**c**) pre- and (**d**) post-treatment.

**Figure 6:**
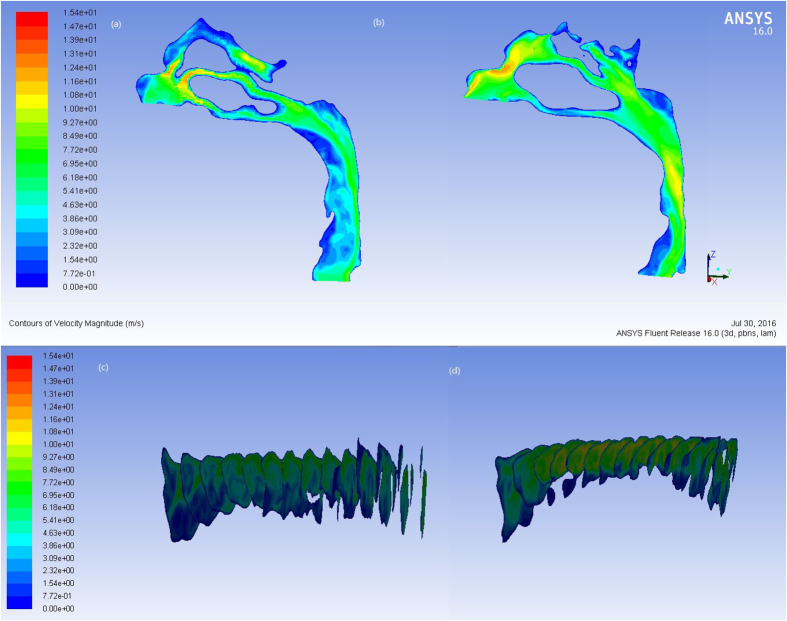
The velocity profile of the pharyngeal airflow in (**a**) pre- and (**b**) post-treatment and the cross section every 5 mm along Z-axis in (**c**) pre- and (**d**) post-treatment.

Figures 5 and 6 showed the pressure and velocity distribution in upper airway for a representative patient. In the pre-treatment subjects, the pressure had no abrupt changes from the choana to the hypopharynx, and the minimum pressure and maximum velocity occurred in the hypopharynx. After extraction treatment, the pressure decreased from the choana to the hypopharynx, and the minimum pressure and maximum velocity occurred in the oropharynx. In addition, the mainstream of airflow with high velocity flowed along the posterior wall of the pharynx, while the flow velocity was lower in the anterior wall. The local swirls occurred mostly in the places with abrupt changes in morphology. The value of the ΔP_max_, Or-ΔP, Hy-ΔP increased significantly after the extraction treatment (Table 1). The pressure drop in nasopharynx was statistically insignificant.

As shown in Table 2, a significant negative correlation was found between Or-ΔP, Hy-ΔP and the relevant volume. There was a significant positive correlation between Or-ΔP, Hy-ΔP, ΔP_max_ and A_min_, A_min_/A_mean_. In Table 3, there was a significant positive correlation between Or-ΔP, Hy-ΔP, ΔP_max_ and ICE.

## Discussion

The present study was designed to assess the airflow characteristics variation of the upper airway in adult class I bimaxillary protrusion patients using functional imaging based on CFD after extraction treatment. The pressure drop from choana to the minimum cross-section, and the resistance in oropharynx and hypopharynx increased significantly in post-treatment subjects compared to the pre-treatment subjects. These changes were correlative with the relevant anatomical changes and the treatment outcomes.

Previous studies have reported a close relationship between pharyngeal narrowing and OSAHS^18^,^19^,^20^,^21^. ΔP_max_ and A_min_ have been proved to be positively correlated with the severity of the OSAS^10^,^20^,^21^, which are parameters to quantify clinically significant flow- and pressure-related effects of anatomical narrowing. These two variables may serve as a guide for clinical treatment. In this study, the correlation between the A_min_ and ΔP_max_ was confirmed. After extraction treatment, the pharyngeal minimum cross section was diminished significantly, which induced additional negative internal pressure and higher airflow velocity due to the “Bernoulli effect” near the smallest cross-sectional area of the upper airway. Bigger pressure gradient between the internal pressure and the external pressure from surrounding tissues made the upper airway a more collapsible vessel. Due to the reduction of the smallest cross-sectional area, pharyngeal luminal pressure decreased more due to an increase in kinetic energy and a dissipative energy loss upstream during inspiration. Consequently, the ΔP_max_ raised significantly, which may suggest higher probability of pharyngeal obstruction. The correlation between ΔP_max_ and A_min_/A_mean_ was stronger than that between A_min_ and ΔP_max_, suggesting that the relative narrowing of the upper airway had a stronger effect on the collapsibility than the absolute narrowing^15^.

In pre-treatment subjects, the airflow passed through the pharynx smoothly without sharp changes. The pressure reduced uniformly from nasopharynx to hypopharynx, and the minimum pressure existed in the hypopharynx. After the extraction treatment, the minimum pressure moved up along the pharynx. This occurred mostly in the root of tongue, next in the back of the soft palate where the pharyngeal obstruction occurred commonly in OSAS patients^22^,^23^,^24^. The resistance of both the oropharynx and hypopharynx raised, which was significantly correlated with the relevant volumetric decrease and the amount of the retraction of the incisors. The retraction of the incisor diminished the length of the oral and enforced the tongue backward, therefore the upper airway diminished in size, especially in the base of the tongue, next in the back of the soft palate^9^. These findings were concordant with Chen Y *et al*.’ studies^6^,^7^,^9^, but not with Al Maaitah *et al*.’ fingings^8^,^24^,^25^, possibly due to the difference in the selected subjects and growth.

It has been demonstrated that the fast changing period of the upper airway is under 20 years old^26^. Since all of the patients in the present study were older than 20, the growth potential impact might be ignored.

The limitation of this study is that the CFD model assumed the wall of the upper airway as hard wall, while the upper airway was surrounded by soft tissues, such as tongue and soft palate in the anterior wall. Therefore, a 3D fluid-structure interaction simulation of the upper airway, which considers the interaction between the airflow and around soft tissues, is needed to assess the functional changes of the upper airway^27^,^28^,^29^. Another limitation is that the OSAS occurs during sleeping, when the upper airway dilator muscles andrespiratory pump muscles are less activated thus cannot adjust the shape and posture of the soft tissues. However, the CBCT has been taken while the patient is awake, and the size and morphology of the upper airway may be different between asleep and awake^30^,^31^. In the future, a FSI model of the sleeping patient should be established to assess the functional changes of the upper airway after the extraction treatment.

## Conclusion

The oropharynx and hypopharynx increase in flow resistance and the risk of collapsing of the pharynx become higher after the extraction treatment with the maximum anchorage in bimaxillary protrusion adult patients. Those adverse changes in upper airway owing to the extraction should be taken into consideration to avoid weakening of the respiratory function.

## Additional Information

**How to cite this article**: Zheng, Z. *et al*. Computational fluid dynamics simulation of the upper airway response to large incisor retraction in adult class I bimaxillary protrusion patients. *Sci. Rep.* **7**, 45706; doi: 10.1038/srep45706 (2017).

**Publisher's note:** Springer Nature remains neutral with regard to jurisdictional claims in published maps and institutional affiliations.

## Figures and Tables

**Table 1:** The parameters measured from 3D models and CFD simulation (Na-nasopharynx, Or-oropharynx, Hy-hypopharynx).

Variables		T1	T2	P	Corrected P
V_Na_(cm^3^)	mean	5.68	5.37	0.677	1.000
	SD	0.59	0.41		
V_Or_(cm^3^)	mean	20.83	15.64	0.009	0.036
	SD	4.86	4.02		
V_Hy_(cm^3^)	mean	8.50	6.04	0.053	0.159
	SD	3.44	2.03		
A_min_(cm^2^)	mean	2.21	1.51	0.006	0.036
	SD	0.64	0.35		
A_min_/A_mean_	mean	0.69	0.97	0.003	0.024
	SD	0.47	0.49		
ΔP_max_(Pa)	mean	33.08	58.93	0.000	0.000
	SD	10.44	24.56		
Na-ΔP (Pa)	mean	13.53	14.88	0.822	1.000
	SD	10.39	9.32		
Or-ΔP (Pa)	mean	19.97	37.43	0.006	0.036
	SD	10.59	16.43		
Hy-ΔP (Pa)	mean	23.80	30.26	0.005	0.036
	SD	13.55	15.77		

**Table 2:** The correlation between pressure drop in minimum cross section, oropharynx, hypopharynx and the relevant morphological parameters.

Variables		V_Or_	V_Hy_	A_min_	A_min_/A_mean_
ΔP_max_	p			0.009	0.000
	r			0.44	0.61
Or-ΔP	p	0.000		0.004	0.005
	r	−0.67		0.68	0.72
Hy-ΔP	p		0.000	0.003	0.004
	r		−0.70	0.44	0.57

**Table 3:** The correlation between pressure drop in minimum cross section, oropharynx, hypopharynx and the amount of incisor retraction.

		ΔP_max_	Or-ΔP	Hy-ΔP
ICE	p	0.003	0.000	0.006
	r	0.79	0.73	0.66
